# Commemorating NIN@100: Empowering the nation through nutrition 1918–2018

**DOI:** 10.1111/mcn.13116

**Published:** 2020-12-21

**Authors:** Thingnganing Longvah, Ananthan Rajendran

**Affiliations:** ^1^ ICMR‐ National Institute of Nutrition, Jamai Osmania PO Hyderabad 500007, TS India

The journey of the National Institute of Nutrition (NIN) began in 1918 from a single room Beri‐Beri Enquiry Unit established by Dr Robert McCarrison at the Pasteur Institute in Conoor, India. After the decline of major epidemic diseases, when the attention shifted to the causal factors of lingering ill health and susceptibility to infectious diseases, the Beri‐Beri Enquiry Unit was transformed into the Deficiency Disease Inquiry Unit in 1925. The Royal Commission on Agriculture in India visited the Deficiency Disease inquiry unit in 1926. McCarrison presented the memorandum on Malnutrition in India to the commission, which helped him put nutrition on the state agenda. As a result, the Deficiency Disease inquiry Unit became the Nutrition Research Laboratories (NRL) in 1929 with Dr McCarrison as its first director. Sir Robert McCarrison retired in 1935, and Dr W. R. Aykroyd succeeded him as the director of the NRL. After World War II, Aykroyd retired, and Dr V. N. Patwardhan took over as the first Indian director of the NRL in 1947. In 1958, the NRL was shifted from Coonoor to Hyderabad based on the Bore Committee recommendation in 1946. The NRL completed 50 years of its existence in 1968 when Dr C. Gopalan was the director, and to mark the Golden Jubilee year, NRL was redesignated as the NIN in 1969. The NIN, through its long and distinguished history, played a significant role in establishing the link between diet and health in the Indian subcontinent. The institute has also monitored the country's food and nutrition situation through the National Nutrition Monitoring Bureau (NNMB) to provide critical inputs to the government to safeguard the population's nutritional status. NIN continues to delve into contemporary nutrition research according to the country's needs to inform the country's health and nutrition policies.

Over the years, India has made remarkable progress in reducing the burden of malnutrition, yet child undernutrition remains unacceptably high while overweight, obesity and nutrition‐related non‐communicable diseases have risen to unprecedented levels. Compounded to that is the millions of people suffering from micronutrient deficiencies. Unhealthy or low‐quality diets are the root cause of all types of malnutrition, including micronutrient deficiencies. Coherent action and innovative food system solutions are needed to ensure access to sustainable, balanced and healthy diets for all. The year 2018 marks the centenary year of the NIN. Therefore, as one of the NIN centenary year milestones, the International Conference on ‘Aligning food systems for healthy diets and improved nutrition’ was organized from November 11–13, 2018. The keynote lecture from ‘food security to nutrition security’ stressed the need to improve food diversity to address the multiple dimensions of malnutrition by bridging agricultural policies with contemporary nutritional challenges. Presenters at the conference plenary sessions examined nutrition and food systems challenges focusing on primary drivers of today's faulty food systems and the need to transform food systems to provide healthy diets against the backdrop of a rapidly changing food systems in the face of urbanization, globalization and trade liberalization. Some presentations examined indigenous food systems' potentials and the need to mainstream neglected potential underutilized indigenous crops for dietary diversification. Against a backdrop of a rapidly growing population, presentations were made on the progress and impact of crop biofortification and/or food fortification to improve nutrient intakes as a public health measure. The conference deliberations also stressed the need to create an enabling environment for enhancing livelihood and promoting a diversified food system to increase the affordability of healthy diets for all through a sustainable food system. A clear message from the conference is that the faulty food systems providing low‐quality diets are the primary drivers of dietary and nutrition inequities. That the multifactorial, interdependent aspects of food systems require a strong evidence base to drive food system transformation policies to provide healthy diets for improved nutrition.

To commemorate the NIN centenary year 2017–2018, some of the plenary and oral presentations made at the second NIN International Conference is being published in this special MCN Supplement issue. We hope you will enjoy reading the papers published in this volume.

